# The Gene-Centric Content Management System and Its Application for Cognitive Proteomics

**DOI:** 10.3390/proteomes6010012

**Published:** 2018-02-23

**Authors:** Ekaterina V. Poverennaya, Alexander V. Shargunov, Elena A. Ponomarenko, Andrey V. Lisitsa

**Affiliations:** Orekhovich Institute of Biomedical Chemistry, Moscow 119191, Russia; torontoxx@yandex.ru (A.V.S.); 2463731@gmail.com (E.A.P.); lisitsa063@gmail.com (A.V.L.)

**Keywords:** knowledge base, data-enabled science, integrative biology, Omics science, Human Proteome Project, chromosome 18

## Abstract

The Human Proteome Project is moving into the next phase of creating and/or reconsidering the functional annotations of proteins using the chromosome-centric paradigm. This challenge cannot be solved exclusively using automated means, but rather requires human intelligence for interpreting the combined data. To foster the integration between human cognition and post-genome array a number of specific tools were recently developed, among them CAPER, GenomewidePDB, and The Proteome Browser (TPB). For the purpose of tackling the task of protein functional annotating the Gene-Centric Content Management System (GenoCMS) was expanded with new features. The goal was to enable bioinformaticans to develop self-made applications and to position these applets within the generalized informational canvas supported by GenoCMS. We report the results of GenoCMS-enabled integration of the concordant informational flows in the chromosome-centric framework of the human chromosome 18 project. The workflow described in the article can be scaled to other human chromosomes, and also supplemented with new tracks created by the user. The GenoCMS is an example of a project-oriented informational system, which are important for public data sharing.

## 1. Introduction

In this paper we report observations from the Chromosome-Centric Proteome Project (C-HPP). The chromosome-centric approach assumes a deep proteomic study of an individual chromosome, which implies the integration of post-genomic data in a geno-centric mode. By default, bioinformatics tools enable us to process the raw data acquired from many of the experiments. At the same time, we witness the birth of new bioinformatics which serve as an integrative technological chain between data acquisition, data processing, and data interpretation.

In view of this, the chromosome-centric approach is key to working within the data-intensive domain [1]. The centricity of the chromosome assists in coping with increasing amounts of data, such as the geometrical growth of the human RNA-seq in the National Center for Biotechnology Information (NCBI) sequence read archive. The volume of the proteome data is increasing more slowly, but still sufficiently—almost twice a year.

In proteome research, meta-analysis of the genome and post-genome data comes to the forefront. Different data sources are interlinked to connect data on protein localization, protein expression, and protein function. The proteins are linked to the proteotypic peptides, as mass-spectrometry (MS) measurements of the peptides remains a method of choice for high-throughput proteome profiling. The initial steps of the Chromosome-Centric Human Proteome Project (C-HPP) have shown the importance of data integrity, in order to be aware of which protein was identified and quantified in which experiment. The value of data integration increases in importance because, for the implementation of C-HPP, the work of the proteome inventory was split among different teams and countries [2].

At the beginning of the C-HPP there were established resources on proteomic MS-data (PRIDE [3], GPMdb [4] and PeptideAtlas [5]); affinity data (Human Protein Atlas [6]); human proteome data in neXtProt [7]; and flexible platforms to work with heterogeneous molecular data, such as TAVERNA [8]. Despite such a representative diversity of existing IT-frameworks, new systems were launched: The Proteome Browser (TBP) [9], GenomewidePDB [10], and CAPER [11]. The core difference of these systems was the sorting of the data in a chromosome-centric way. However, further deep mining of the human proteome has shown that each large-scale experiment unavoidably delivered a new informational resource, most notably those created due to the outstanding work of the scientific groups lead by Mathias Mann [12], Bernhard Kuster [13], Mathias Uhlén [14], and others.

The Russian part of the C-HPP also needed to develop a specific resource. This need was mostly due to the requirement to couple the phases of the research process. To analyze the proteomic complement of human chromosome 18 (Chr18) in three types of biomaterial (blood plasma, tissue, and cell line) a special knowledgebase has been developed; the kb18.ru [15,16]. The kb18.ru is an example of implementation of the GenoCMS Web-Platform.

Here we describe the features and applications of kb18.ru/GenoCMS to give a rationale for project-driven bioinformatics. We demonstrate that the creation of the IT-environment is driven not only by the aim of presenting the results, but also by the necessity of serving the highly specific process of organization of the work of the research consortia.

## 2. Materials and Methods

### 2.1. Data Sources

The GenoCMS was assembled using the UniProt identifiers of the proteins. The list of the protein products of Chr18 was automatically retrieved through the UniProt search interface. The list was updated each month to maintain synchronization with UniProt, and also with neXtProt.

The data for the proteins of Chr18 was organized into several domains. The data for first domain was directly uploaded from the public databases, while the second was created using algorithms for text-mining of scientific articles in PubMed. The third domain was supported by manual inspection of the supplementary materials of the articles reporting sufficient coverage of the proteome by bottom-up MS. Finally, the fourth domain was created using the results of the proprietary experiments, which were performed by the Russian part of the C-HPP [17,18,19].

The GenoCMS interlinks the information from the dynamically-linked public resources by using the Neosematic/RESTful BioCurations technology (ProContent Ltd., Kleinmachnow, Germany). The dynamic link was supported by the flexible programming interface, enabling on-line updating of the information. This means that within the GenoCMS each interface script was embedded into a certain schedule to upload new data periodically. Doing so required developing typical patterns for the requested incoming data, in order to observe whether the format of the queries sent to the public resources remained unchanged.

For the public molecular informatics resources, we have selected the following (this particular set of data sources is dynamically adjusted by the GenoCMS users): information about proteins and genes of Chr18 was taken from UniProt and neXtProt, and the data was loaded from the gene expression banks (GEA, GTEx, and RNAseqAtlas); mass-spectrometry and affinity proteomics (GPMdb, PRIDE, PeptideAtlas, ProteinAtlas); interactomics (IntAct, BioGRID, and others); and from disease-related databases, like OMIM and GeneCards. For the other origins of publicly available data utilized in this paper, refer to the description of the GenoCMS [15,16].

### 2.2. Data Management

**Figure 1:**
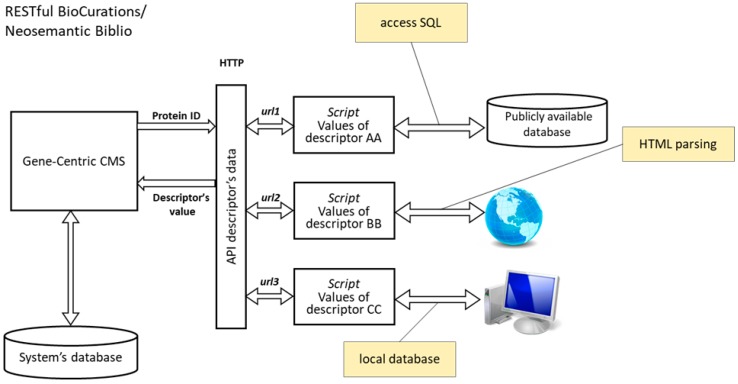
Data management within the Gene-Centric Content Management System. Descriptors are used to bind the local flow of the proteomics data to the algorithmically-derived data and the data loaded from the public resources on molecular biology. url—universal resource locator, supporting the RESTful protocol.

GenoCMS is based on descriptors (e.g., particular concentrations of protein X in the tissue Y, measured by different methods) which are combined into data tracks (e.g., the presence of the protein amongst all of the tissues, or vice-a-versa, abundance of the proteins X_1_, Х_2_ … Х_n_ in tissue Y). The core of the system is presented by the web-based heat-matrix, which serves as a hub to link databases and manual or automated requests, driven via the programming interface (API) (see Figure 1).

The GenoCMS arranges the data in the form of a heat matrix, where raw proteins (or genes) and columns are the descriptors. The value of the descriptor is quantified according to the volume of the available data on the protein. The data is retrieved either from the international public databases, from the text-parsing of the web-pages and collections of PubMed articles, or from the local database server (Figure 1). The local server of the Chr18 part of the Russian HPP contains the results of more than 5500 measurements made by selected reaction monitoring mass-spectrometry (SRM-MS) and shotgun mass spectrometry (MS)/MS for human chromosome 18. Communications between the interactive core of the GenoCMS (heat-map) and the data sources were organized by the URL protocol using the RESTful Biocurations platform.

## 3. Results

### 3.1. The Workflow

**Figure 2:**
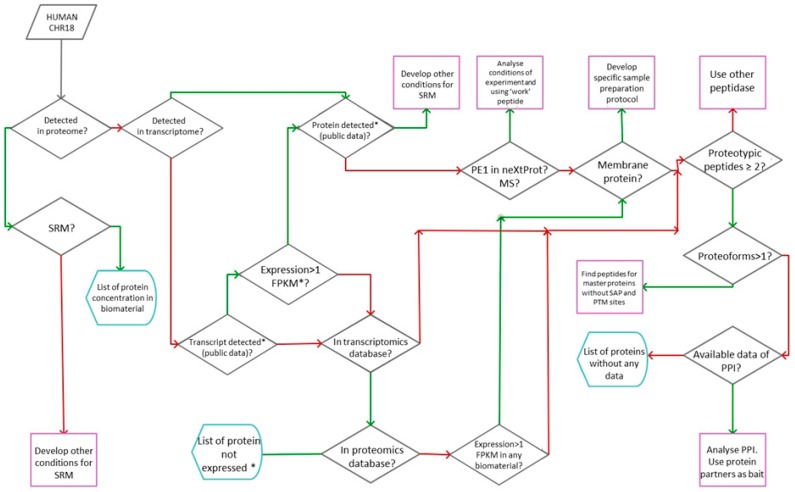
Sort-and-select workflow for planning and implementing the chromosome-centric analysis (using the example of Chr18). A red arrows denotes “NO”, a green arrows denote «YES». See text for explanation. * in the target biomaterial, i.e., liver tissue/HwpG2 cell line/human blood plasma.

In Figure 2 we present the general workflow as a result of the implementation of the Russian part of the Chromosome-Centric Human Proteome Project, chromosome 18. To implement the workflow we have developed the GenoCMS—the Gene-Centric Content Management System.

The GenoCMS, coupled with the efforts of several laboratories, provided the framework. The Laboratory of Personalized Medicine helped in collecting and processing the data on the relevance of links between genes and diseases. The Postgenome Analytics Laboratory contributed by developing the tiny software scripts, which automatically uploaded the information on chromosome 18 into the GenoCMS. The information was linked, then, to over 35 resources, including neXtProt, NCBI Sequence Read Archive, and many others. Next, the Laboratory of Systems Biology shared the raw data on the proteotypic peptides for Chr18 coded proteins. These peptides were synthesized as isotopically-labelled standards (SIS), and measured for the three relevant types of biomaterial (blood plasma, cell line, and tissue). Finally, in collaboration with two laboratories with RNA-seq technology, the data was obtained on exomes for the cell line and for the tissue samples.

Integration of the whole data array was performed by the Postgenome Analytics Laboratory using the previously reported features of the GenoCMS [15,16]. The details of the data assembly are shown in Figure 2.

The input of the GenoCMS included the initial knowledge of human chromosome 18; information was fetched from UniProt as a list of gene identifiers (see Figure 2). For each gene, whether or not the proteins were detected in our proprietary set of experiments was deciphered [17,18,19,20]. Within the set it was necessary to sort out which genes were detected at the protein level by SRM-MS and which of them were expressed at the transcriptome level (detected by NGS—next generation sequencing).

Following the scheme in Figure 2, the next stage was comparing the proprietary results to external databases. If the protein was not observed by SRM, the GenoCMS armed the coordinator of the project with other sources for making decisions. For example, the gene was searched in the transcriptomic and proteomics databases for the number of entries in neXtProt.

An example to consider is if a given gene is expressed with a high FPKM-value (fragments per kilobase of transcript per million mapped reads), has a Protein Existence according the neXtProt (PE), and was also observed in a significant number of the proteomics experiments in PRIDE. However, no significant SRM-signal was observed either in blood, in the HepG2 cells, or in the human liver tissue. This means that within the GenoCMS such genes are “re-posted” for the design and synthesis of the next batch of the proteotypic peptide, which reinforces the need for the development of updated versions of the SRM-method.

Similarly, by sorting and selecting the information deposited in the GenoCMS it was possible to derive practical advice regarding:

(a) adjusting the method of SRM analysis for a given peptide;
(b) synthesizing new SIS peptides for a certain protein;
(c) problems in the sample preparation protocol (e.g., the target protein is membrane-bound);
(d) the value of using another peptidase (to achieve better coverage for an interesting protein).

The rightmost part of the scheme in Figure 2 illustrates that the GenoCMS could be sensitive to rare events such as single amino-acid modifications and single amino-acid polymorphisms (SAPs/SAAPs). In some cases the transcript and protein were both missing in the current databases, however in proprietary experiments the gene transcription was reported at a relatively high level (at least RPKM >1). Such genes were selected as potential candidates to search for the functionally important proteoforms [21,22].

### 3.2. Parsing the Selected Datasets

**Figure 3:**
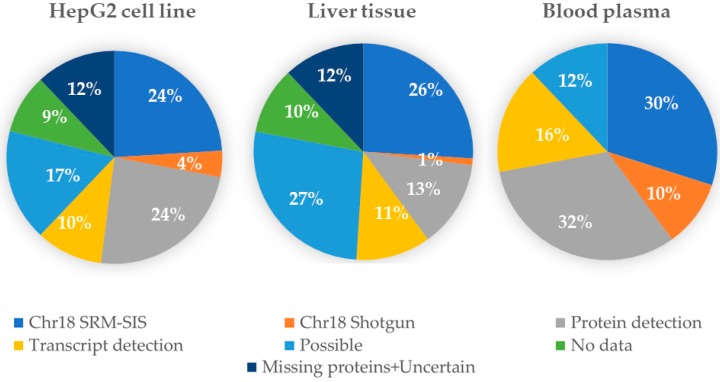
Using the GenoCMS dataset as a bioinformatics radar for the Chr18 protein detection/identification by targeted SRM (Chr18 SRM-SIS) or by the MS/MS (Chr18 Shotgun) methods across three types of biomaterial with linkage to the types of evidence, taken from the neXtProt resource.

After collecting the results, the GenoCMS was used for statistical monitoring of the Chr18 data. The dataset, with specific descriptors, was used to create the diagram shown in Figure 3. Using this dataset it was possible to observe that, independently of the type of biomaterial, 30–40% of the proteins encoded by Chr18 are easily retrieved by MS. The dataset also contained, for the same types of biomaterial, the publicly deposited results of protein identification. Therefore, while our intrinsic experiments delivered at maximum 40% Chr18 coverage, by appending the data from the public resources chromosome coverage of 63% was achieved.

Currently, then, the GenoCMS maintains the benchmark of Chr18 at the level of 63%, leaving 37% leftover for the so-called missing proteins. The degree of chromosome coverage confirmed by the GenoCMS tool is lower than the data published by the leading groups [13,23], and is also sufficiently lower than the coverage claimed in the previous research of Chr18 in the Russian part of the C-HPP [18,19,20,24]. This means that local content management enables us to perform the internal assessment of the validity of the results of the proteome analysis.

The CMS approach forces researchers to follow the principle of an open biodata supporting the versions of the datasets. In comparing the versions, it was possible to observe the dynamics of the results and to highlight some details in Figure 3. For example, 32% of Chr18 proteins were detected in plasma, and 24% in the HepG2 cell line. However, half of that number of proteins (13%) were detected in the liver tissue. This proportion remains unchanged for the datasets of different years, starting from 2014, despite the noticeable improvement of the quality of the MS-measurements. Does this mean that HepG2 is drastically different from the liver? Could we expect that blood generally collects significantly more proteins than are produced by the liver cells? Such disputable observations potentially lead to the hypotheses which comprise an essential outcome from the cognitive application of the GenoCMS.

### 3.3. Missing Because of Splicing?

**Figure 4:**
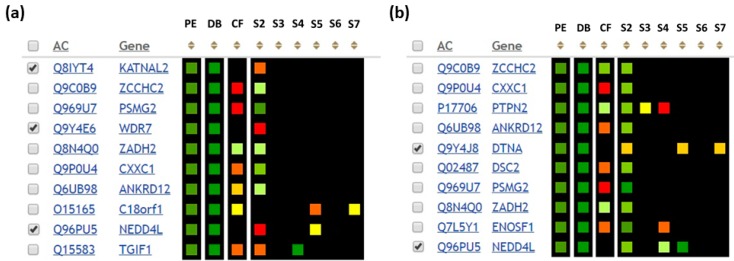
Datasets to map the canonical and spliced forms of missing proteins of Chr18 in the liver tissue (**a**) and in the HepG2 cell line (**b**). PE: protein evidence according to neXtProt; DB: information from several mass-spectrometry databases on protein detection in the biosample; Chr18 HPP transcriptomic data [19,20,26]; CF: level of expression of canonical form; S2–S7: levels of expression of the splice forms. Colored boxes represent the quantitative value assigned to the descriptor. The color code indicates the amount of information: a black box indicates the absence of data, a red box indicates relatively little data, while a green box indicates sufficient data. This particular color scheme was developed by the kb18-user, who is the owner of the descriptor. The appropriate kb18-datasets are accessible at http:/*/426511 for the liver tissue, and at http:/*/426516 for the HepG2 cells. *kb18.ru/protein/matrix.

Using the GenoCMS we have analyzed the missing proteins of Chr18 [25]. In browsing the kaleidoscope of heat-matrices of different datasets it was noticed that sometimes missing proteins become visible (see Figure 4). These appearances were due to the transcriptome profiling of the cell line HepG2 and the liver tissue. It was found that there were no expressions of the canonical form but expression of splice forms S2–S7, as shown at Figure 4, for three and two genes respectively, (Figure 4a) and HepG2 (Figure 4b), was observed in the liver tissue. Visually this is shown in Figure 4 as a black background color in column CF (canonical form), and colored squares for the S2–S7 descriptors (the color depends on the level of expression of the splice variant).

The situation shown in Figure 4 highlighted the importance of observing the splice variants in the framework of the chromosome-centric HPP. In particular, in comparing the datasets presented in Figure 4a,b, it is possible to identify the NEDD4-like gene, where NEDD is the neural precursor cell expressed developmentally downregulated gene. This gene is not observed in a canonical form, but is represented by several splice forms both in the tissue and in the cell line.

Therefore, the GenoCMS helped to locate the proteins not identified by the proteome analysis (so-called missing proteins), which may have occurred due to their possible expression as a splice form. To tackle some primary targets for development of the SRM assay, it was helpful to create the tissue-dependent map of splicing by programming the specific descriptors S2–S7 (Figure 4). For further planning it was reasonable to recommend the use of HepG2 as the biomaterial and to develop the SIS proteotypic peptide to the spliced form of the missing protein product of the gene NEDD4L.

### 3.4. Guilt by Association

During HUPO (The Human Proteome Organization) Congress in 2017 the next vector of the Human Proteome Project was proposed in order to achieve deeper functional annotation of genes. The primary focus is on the genes which lack functional annotation, although they were detected at the proteome level according to neXtProt. It was also discussed that gene editing technology would influence the roadmap of C-HPP in a way that would enable the exploration of the changes of the proteome after corrupting or enhancing the translation of the genes of the targeted chromosome.

**Figure 5:**
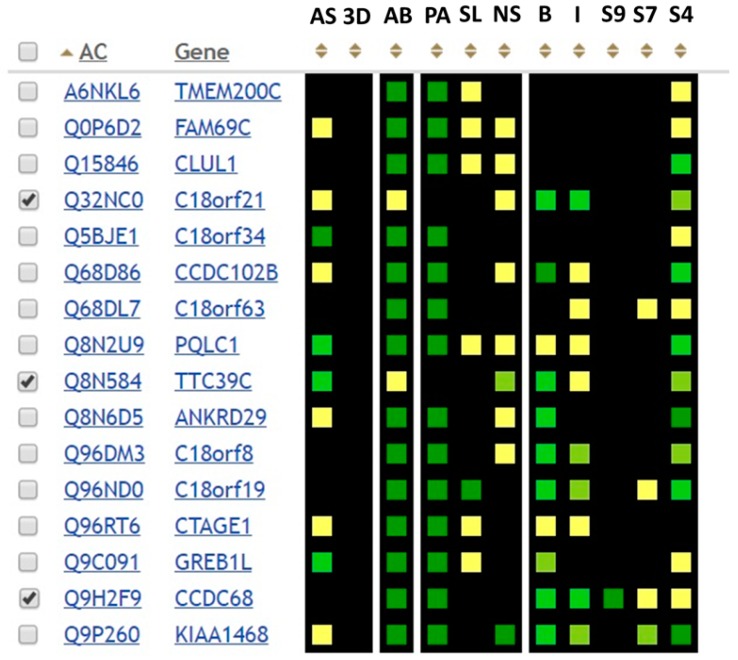
The dataset for Chr18 proteins of unknown function. AS is the number of splice-forms (isoforms) according to the neXtProt; 3D is the data on the 3D structure (PDB); AB is the availability of the antibodies (Antibodypedia/Human Protein Atlas); PA is the number of different tissues where the gene product was detected by MS (PeptideAltas and PRIDE); SL is the subcellular localization according to the Gene Ontology; NS is the number of isoforms predicted for different tissues; B is the number of interactions according to the BioGRID; I is the number of interactions according to IntACT; S9–S4 is the number of interactions according to StringDB, S9 is the score >0.9; S7 is the score >0.7; S4 is the score >0.4. Colored boxes represent the quantitative value assigned to the descriptor. The color code indicates the amount of information: a black box indicates the absence of data, a yellow box indicates relatively little data, while a green box indicates sufficient data. The described color scheme is automatically counted according to 20% thresholds. The kb18-dataset is available at http://kb18.ru/protein/matrix/426530.

The GenoCMS helped us to assemble the dataset of putative targets for functional annotation (Figure 5). There were a total of 16 proteins on the list, which were observed either using SRM or MS/MS with a strong level of confidence. Five of them are named according to the identifiers of open reading frames (ORF).

Their existence at the proteome level, which was detected during the Chr18-centric HPP, was confirmed by two other data sources. First, in paying attention to the descriptor “AB”, one can see the green squares in the respective columns for almost all of the 16 proteins. This means that antibodies are available, and the green color of the square convinces us that most of the antibodies are trustworthy. Secondly, with the exception of two proteins, all of the other proteins were deposited into the global MS databases. The mass-spectrometry data was obtained independently from different tissues (the color of the descriptor is proportional to the number of MS records containing meta-data on the tissue type).

Approximately half of the entries of our sample had some information about the localization of the protein in the cell (see the SL descriptor in Figure 5). The yellow color indicates a moderate degree of confidence, in that there could be some specificity of subcellular localization. Therefore, in general, cell fractionation is not the method of choice to explore the functional roles of the target proteins, with the exception of C18orf19—the 30.1 kDa mitochondrial protein of unknown function.

To exemplify the cognitive proteomics, an idea of “guilt-by-association” was revisited. The visual perception of the dataset delivered the next two targets, namely C18orf21 and TTC39C. The cognitive association was that both proteins were detected in the liver tissue and the HepG2 cells in our experiments, and both were missing from the world-wide databases of mass-spectrometry data. Each protein was interesting in terms of functional annotation. C18orf21 had a low signal for the presence of the antibody, and a high or sufficient signal in the block of interactomic descriptors (BioGRID, IntAct, and StringDB). TTC39C also had low AB-signal comparable to the degree of involvement in the molecular interactions, but strong evidence for existence of splice-forms (Figure 5).

Following the rule of guilt-by-association one more target could be proposed; the ССВС68 protein. Contrary to the previous two targets (C18orf21 and TTC39C), we observe strong evidence that CCBC68 was detected in many tissues by MS and AB. The “guilt” of CCBC68 was that it demonstrated the highest degree of occurrence in the interactome databases (see columns B, I, and S9–S7 in Figure 5). It was the only protein which achieved a green color in the StringDB with an extremely high score of >0.9.

## 4. Discussion

The GenoCMS was used to implement the kb18.ru for data storage and data sharing, but was also used as an example of a web-based system enveloping proteins by cognitive thinking. The targets for functional annotations (NEDD4L, C18orf21, TTC39C, and CCBC68) were selected because of the intuitive personal perception of the data shown in Figure 5, without stringent statistical justification. For the web-development, the content management systems provide basic modules which are used to represent content in a smartly designed and cognitively attractive mode. The same modules are used to present such diverse elements as fitness clubs, sales or painting services, or any other service delivered through the Internet. Despite such systems being quite different in implementation, all of them share the same relevant feature: a successful CMS provides a transformation of the coordinated multi-individual process into a result which is consumed by the wide audience of end-users. We observed that the principle of data-enabled process coordination is essential for the implementation of the large-scale international initiative, the C-HPP.

In hacking the human proteome in a chromosome-centric way, non-obtrusive coordination becomes even more valuable than the final result itself. This is confirmed by papers reporting home-made systems; every significant result required a specific software environment for appropriate assembly and convenient representation [13,27].

To understand the phenomenon of home-made resources we inquired regarding the raw data of the recent large affinity-purification mass spectrometry (AP-MS) experiment [27]; the provision of the raw experiments was guaranteed in the publication. Communication between the data requester and the authors confirmed that all of the necessary information was available from the home-made proprietary resource. It seems that the project-oriented databases (local depositaries) are increasing in importance, because they deliver cutting edge research.

What usually makes the leading investigators choose to preserve these data locally, instead of uploading into the public domain? Superficially, the data is preserved because of the potential usefulness for the data producer. In our view, there is another significant reason to share not through the public resources, but instead via local databases. That is, it is an IT-development in the domain of data, on how to organize the process of generation of the data we described in the gene-centric workflow, where each block was implemented by one of the teams participating in the Chr18 proteome project.

Experience with the development and exploitation of the GenoCMS since 2011 convinced us that post-genome projects were doomed to be supported by home-made data/content management systems. Up until now, there was no justified reason to develop “one-for-all” gene-based software environments. Contrary to the universal platform, for the foreseeable future every project in post-genome science (including proteomics) should provide for the expense of custom software development.

To support this view we shared the specific cases of interaction of the user with the GenoCMS for extraction of the working hypothesis. The GenoCMS was used for the practical purpose of finding which tissue can be used to detect the missing protein.

Working within the GenoCMS (using heat-maps and sort-and-select workflows) it was observed that proteoforms contribute a reasonable distortion into the process of detection and identification of proteins [28]. Information about transcriptional/translational and post-translational aberrations comprise an important source of disease-related information, but creates difficulties for MS-detection [29,30]. Combining the different data sources allowed us to propose the less obvious peptides for the SRM experiments, targeted to the missing proteins.

We have ranked the peptides in accordance with their susceptibility to single amino-acid polymorphisms (SAPs/SAAPs) and post-translational modifications. In our statistics, accounting for data in proteoforms is essential for at least half of the missing proteins coded by the genes of Chr18.

Since the diversity of human proteins reaches several million different proteoforms according to some estimates [31], in the future a peptide-centric approach of data analysis could be used in the functional annotation of proteoforms. Further development of the current resource involves new characteristics (descriptors) of genes linked to new resources based on the described system for other chromosomes and peptides.

## 5. Conclusions

Currently there is the lack of a resource which could be universally useful for describing an abstract proteomic experiment. Moreover, it is not evident that such universal tool would be helpful for increasing the knowledge of the human genome. We assume that to decipher the genome at the proteome level, small separate solutions would be as relevant as large platforms, through absorbing all of the experimental data.

The question to consider is whether it is worthwhile to build yet another bioinformatics system for integration of the post-genome data. Because each and every large-scale post-genome project inevitably required the creation of a separate resource, Human Protein Atlas or PeptideAtlas, C-HPP resources, or other domestic products of the same class, the answer to this is undoubtedly yes.

Faced by the novel, counterintuitive chromosome-centric format, we developed the Gene-Centric Content Management System (GenoCMS). The system is based on the application of common Internet protocols, which are affordably programmable (even by students). In our opinion, such an open architecture would foster the functional annotation of the proteins (additional to crowdsourcing).

Using the GenoCMS as a prototype environment it was demonstrated that proteoforms are distinguishable entities. It should be noted that bottom-up mass-spectrometry, due to technical limitations, generated a quasi-realistic (or possibly unrealistic) view on the breadth of the human proteome [31]. By thoroughly integrating diversified information it could be possible to uncover the real world proteoforms, produced due to genome polymorphism, RNA-processing and editing, and natural chemical modifications.

We conclude that development of local bioinformatics resources enhances the cognitive power invested into proteome science. These resources would very rarely maturate into well-established ones; however, this does not diminish their value. Home-made resources preserve the design of the experiment in a programmatically formalized manner. The necessary prerequisite for extracting added value from the proprietary web-based bioinformatics is a high density of links to core resources. In kb18.ru we achieved that by allowing interface scripts, which act as specific binders between local data and the informational array.

The use of other protein-coding genes as objects for the kb18 system allows the creation of a new gene-centric knowledgebase for other chromosomes of groups of interesting genes, and even peptide-centric systems.
